# Health Tract, No. 84

**Published:** 1862-07

**Authors:** 


### HEALTH TRACT, No. 84.

---

### CORN BREAD.

Hay, straw, fodder, etc., are what farriers call "roughness" to horses and cattle, as compared with a diet of oats or corn alone. Horses kept in the stable and fed on oats, soon become feverish and "bound," and unless relieved will die. Rough food, that is, hay and fodder, are the remedies. So coarse corn-meal made into bread, cakes, pies, pudding, etc., are the "roughness" as compared with eating the various preparations of fine flour. Many persons would be relieved of internal fevers, constipation, indigestion, and other similar ailments, if wheat-flour was discarded in whole, or at least in large part, and corn-bread with various corn-meal preparations were used instead, at every meal. It is generally thought that the corn-bread of the East is never so good as the corn-bread met with on Western tables. The chief reason perhaps is that in the East the corn is ground too fine, and there is something due to skill in baking. There are so many ways of cooking corn-meal, so many modes in which it can be served up on the table, that a person need not get tired of it for months in succession. Mrs. James O'Brien, of Carrick, Pennsylvania, makes her celebrated corn-bread thus: To two quarts of meal add one pint of bread-sponge; water sufficient to wet the whole; add half a pint of flour and a table-spoonful of salt; let it rise; then knead well for the second time, place the dough in the oven, and allow it to bake an hour and a half.

Corn Griddle-Cake.—Scald at night half the quantity of meal to be used; mix the other with cold water until it is a thick batter; add a little salt and set it to rise without yeast. This will make light, crisp cakes in the morning. The skimmings of boiled meat is the best to fry them with. Fry slowly.

Corn-Meal Pudding.—Cool one quart of mush with nearly as much new sweet milk, add five eggs, half a teacupful of sugar, one teacupful of flour, a little salt and quick yeast; bake one hour in moderately slow oven, and eat with sauce, or butter if no sugar is used.

Corn-Meal Pies are made by Mary Williams, of Linn Co., Iowa, thus: Stir a small teacupful of very fine ground Indian meal into two quarts of boiling milk; when nearly cool add five beaten eggs, and sweeten to taste, like a custard, adding spice and orange-peel if desired. Bake with a crust like custard-pies.

Old-Fashioned Hulled Corn.—Shell a dozen ears of ripe, dry corn, put it in an iron kettle and cover with cold water; put in the corn a bag of two teacupfuls of fresh wood-ashes, and boil until the corn looks yellow and tastes strong of the alkali, then take out the bag and boil the corn in the lye over an hour, then pour off the lye, add fresh water and simmer until the corn swells. If the hulls do not then come off by stirring, turn off the water and rub them off with a towel; add more water and simmer for three or four hours, often stirring to keep it from burning; when it swells out and becomes soft and white, add salt to liking, and let all the water simmer away. Eat warm or cold with cream or milk. All these receipts require practice, skill, observation, and judgment. Mix two parts of new corn-meal with three parts of warm water, add one teaspoonful of salt, two teaspoonfuls of sugar, one large tablespoonful of hop-yeast; after rising five hours add three fourths of a pint of wheat-flour and half a pint of warm water; let it rise again for an hour and a half; pour it into a well-greased pan, let it rise a few minutes; let bake an hour and twenty minutes in a moderately hot oven.

Corn Sweet-Cake.—Rub well together a teacupful of butter with two of sugar; five eggs, the whites beaten apart, one cup of sour milk, three of corn-meal, two of wheat-flour, half a nutmeg, with yeast enough to make it rise.

Family Indian Loaf.—Two quarts scalding hot skim-milk, one tablespoonful salt, one quart corn-meal, stirred in by handfuls, two thirds pint of sifted rye-meal, stir thoroughly, then add one cup of cold milk, stirring smartly; after standing twelve minutes, bake five hours in a cast-iron basin, covered with another basin.

